# Accident Risk in the Production Sector of EU Countries—Cohort Studies

**DOI:** 10.3390/ijerph18073618

**Published:** 2021-03-31

**Authors:** Krzysztof Nowacki

**Affiliations:** Department of Production Engineering, Silesian University of Technology, 44-100 Katowice, Poland; krzysztof.nowacki@polsl.pl

**Keywords:** relative risk, accidents at work, production, European Union, GDP

## Abstract

(1) Background: accident rates prove the uneven development of the member countries in the area of work safety. Remedial actions and structural programmes should take into account, e.g., the level of work safety in all European Union (EU) countries. Aim: the identification of differences in the level of work safety in the production sector of EU countries, especially the so-called “old” and “new” EU countries. (2) Methods: for each country UE (in 2008–2018), the relative risk (RR) of an accident at work was determined and a comparative analysis was conducted. (3) Results: an increase in the RR of an accident at work was observed along with an increase in the GDP of a given country. It was found that the level of occupational safety in Sweden and the United Kingdom is higher than in other countries, and lower in Spain and Portugal. In the three largest economies of the EU, Germany, France, and Italy, the RR of the accident in the industrial sector in relation to the national data is one of the lowest in the entire EU, not exceeding 1.3. In The Netherlands, an increase of 1.7 RR of fatal accidents in the industrial sector was observed between 2008 and 2018. (4) Conclusions: RR in the manufacturing sector of the so-called “old” EU is higher than in the so-called “new” EU, which may result from the implementation of Industry 4.0 assumptions in the “old” EU. The presented results and conclusions may be useful in shaping the EU policy in the field of sustainable development of production sectors of individual member countries.

## 1. Introduction

The European Commission invests in European Union (EU) industry for a modern, clean, and fair economy. By promoting the competitiveness of industry through a number of initiatives related to, e.g., the Horizon 2020 program [1], it aims to empower citizens and possess the best technologies for the smart, innovative industry of the future. The countries of the European Union consisting of 27 countries (GB withdrew from the EU in 2020) together have, after the USA, the second highest GDP in the world in terms of per capita income. Countries of the so-called “old” Union, which is, forming the EU before the accession of new members in 2004, are in the forefront of the world, and some regions of Europe have almost twice the level of GDP than the average for all member countries [2,3,4].

The share of industrial production (excluding construction) accounts for around 20% of the total gross value that is added in the EU. Only broad categories of economic activity, defined by Eurostat as wholesale and retail trade, transport, accommodation, and catering services, and public administration, defence, education, health care, and social work, have a similar share [5]. In the EU, there is a high concentration of industrial production in five economies—Germany, Italy, France, Great Britain (until 2019), and Spain—generated nearly 75% of the total gross value added of industrial production. Almost 70% of Europeans working in manufacturing were concentrated in Germany, Italy, France, Great Britain, and Poland [5].

The results that were published in the literature indicate multidirectional research on the risk of work-related accidents in various industries and countries. The research took into account both professional and non-professional factors influencing the increased risk of an accident. Research [6] found that work overload is related to the adoption of risky behaviours, and this fact may have an impact on the occurrence and severity of accidents. The disproportion in the incidence of accidents at work, road accidents, home and sports accidents, as well as the role of conditions and lifestyle is examined, in particular factors such as smoking, excessive alcohol consumption, obesity, taking psychotropic drugs, and disability, influence the risk of an accident [7,8,9]. It was found that work, gender, young age, smoking addiction, alcohol abuse, overweight, psychotropic drug use, and illness had an influence on accidents at work [10]. A frequent subject of research is demographic factors influencing the risk of accidents at work, e.g., organisational and personal factors that underlie the safety behaviour of older construction workers [11], or risk studies related to fatal accidents [12]. An important factor is also the job seniority in the company, which reduces the risk of an accident at work [13]. Accident-prone work that increases the risk of an accident is night work, the effect of which is similar to jet lag [14]. Because there are indications that sleep disorders are related to accidents at work [15], it was investigated how the accident risk increased with successive night shifts, with increasing working hours and between successive rest breaks.

In European labour market analyses, an accident is defined as a sudden occurrence at work that causes physical or mental injury. Accidents that result in an inability to work longer than three calendar days are registered, excluding the day of the accident at work, and fatal accidents are those that resulted in the death of the employee within one year of the incident. An accident at work is any event related to work and that took place on the premises of the workplace as well as outside of it, e.g., accidents in public transport [16]. Data on accidents at work are often published in the form of generally accepted indicators, which are used by statistical offices as part of the analysis of the state of work safety in a country or industry. For example, in Poland, according to CSO (Central Statistical Office) data, in the first quarter of 2020, the highest accident rate per 1,000 employees was recorded in the following industries: mining and excavation (indicator 3.0); water supply, sewage and waste management (2.5); and, healthcare and social work (1.75) [17]. In addition, there are a number of other indicators or accident models [18] which analyse available data on accidents, a specific industry, size of enterprises, age of employees, job seniority, hours worked, etc. Data on accidents, their type, and consequences are also analysed in the European Union, e.g., by the European Statistical Office—Eurostat.

Because of the changing number of people employed in the sector and throughout the EU, as well as the changing number of people injured in accidents, including fatal accidents, there are no clear results indicating the dynamics of changes in the area of work safety in individual EU member countries. This type of indicator, independent of the above changes, is the so-called relative risk (RR), which, when compared to the EU or national level, allows for the assessment of the sustainable development of the member countries in the area of work safety. The aim of the research was to identify differences in the level of work safety in the production sector of EU countries.

## 2. Methods and Research Results

The research material consisted of statistical data on the number of people injured in accidents at work and the number of employees in the European Union countries. Data are collected by Eurostat [5]. In order to determine the trend of possible changes or repeatability of the safety level, data from 2008–2018 were used for the analysis. Because of the methodology of collecting data by Eurostat, 2018 is currently the last year with complete accident data (except for France). The data concerned the summary statement and for the EU production sector. The data at the end of the fourth quarter of each year were adopted as the number of employees in each case.

For each country and year, the relative risk (RR) of an accident at work was determined. The number of accidents in the EU was taken as a reference in relation to the number of people employed in the EU or in the production sector in the EU. The RR was determined by Equation (1)

(1) RR=Q11Q11+Q12Q21Q21+Q22

where:

*Q*_ij_—multiplicity observed in the contingency table (i—column; j—row in contingency table, in this case, a two-way table)

The following hypothesis was adopted: null hypothesis that H_0_: RR = 1 and the alternative hypothesis that H_1_: RR ≠ 1. To verify the hypothesis that the risk of occurrence of the phenomenon under study is the same in the exposed group and in the group not exposed to the risk factor, the test statistic was expressed as (2)

(2) $$Z=\frac{\ln\left(RR\right)}{SE}$$

where:

$SE=\sqrt{\frac{1}{Q_{11}}-\frac{1}{Q_{11}+Q_{12}}+\frac{1}{Q_{21}}-\frac{1}{Q_{21}+Q_{22}}}$—standard error of the logarithm RR

When determining the p parameter, the value ∝ = 0.05 was each time set. Because the data covered different countries, a common effect was presented for the collected data by designating a variable effect, which is due to the fact that each study represents a slightly different population (country), so the real (population) effect will be different for each population. As each study concerned a different country, in order to summarise the meta-analysis with a variable effect, it was assumed that some factors that could distort the size of this effect may have different sizes in individual countries. The data were subjected to meta-analysis determining, due to the cohort nature of the research, for individual years and for the entire analysed period, as the final effect, the relative risk (RR) related to the occurrence of an accident at work in relation to the analysed range. The analyses were carried out in MS Excel 2019 (Microsoft, Redmond, WA, USA) and PQstat v 1.6.8.384 (PQStat Software, Poznań, Poland).

**Figure 1:**
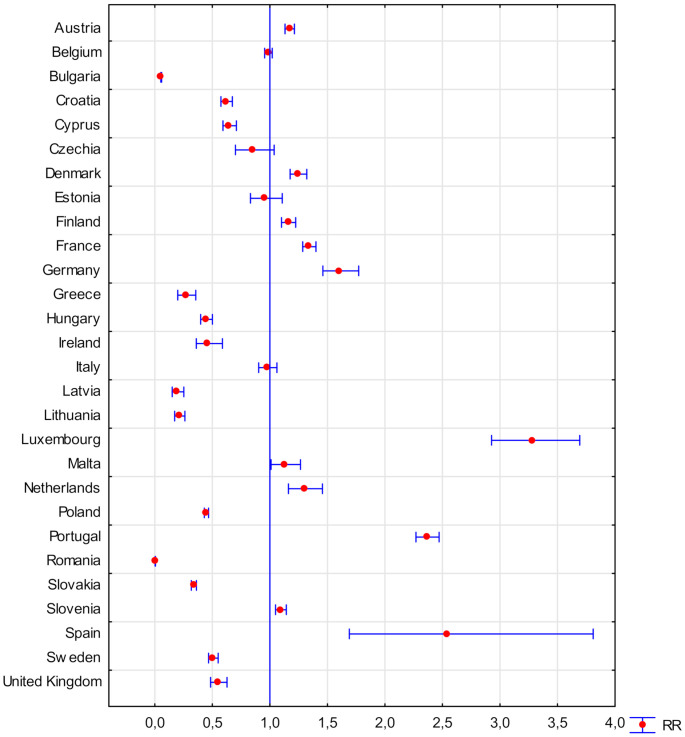
Relative risk of an accident at work in the production sector in European Union (EU) countries in relation to the risk in the EU industrial sector in 2008–2018 (own elaboration).

**Figure 2:**
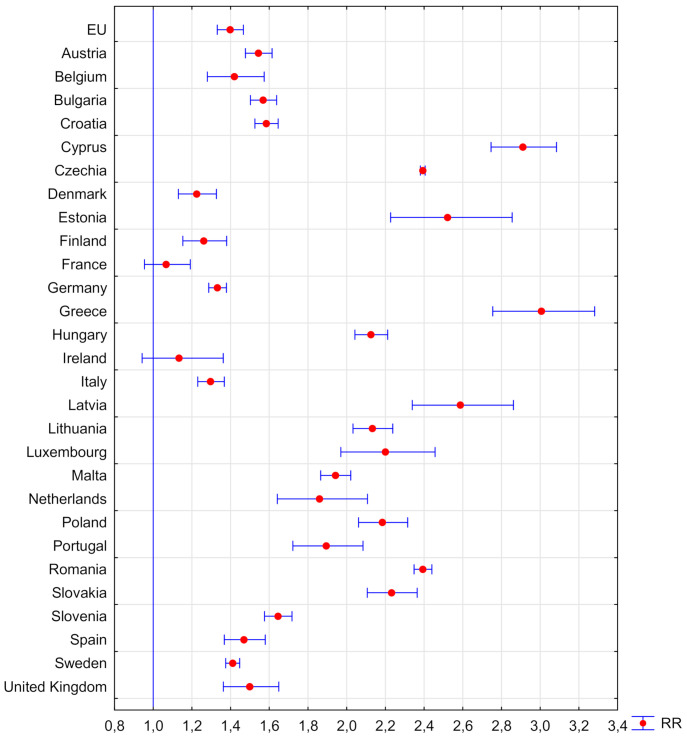
Relative risk of an accident at work in the production sector of the EU countries in relation to the risk of an accident at work in the country in the years 2008–2018 (own elaboration).

**Figure 3:**
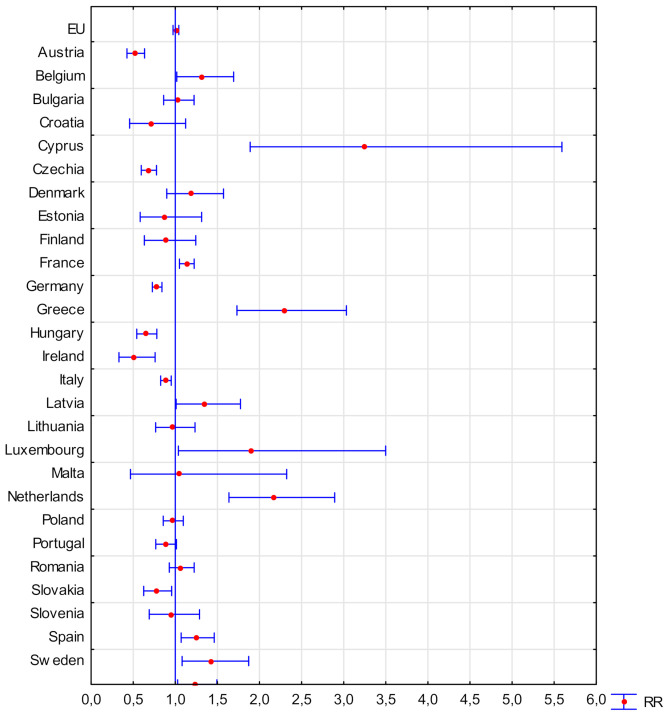
Relative risk of a fatal accident at work in the production sector in the EU countries in relation to the risk of a fatal accident at work in the country in 2008–2018 (own elaboration).

Table 1 shows the RR results for the whole of the EU, and Figure 1, Figure 2 and Figure 3 for the manufacturing sector in the EU. 

## 3. Discussion of the Results

The absolute numbers of registered accidents at work in the European Union countries indicate that, in 2018, as compared to 2008, most countries recorded a decrease in the number of people that were injured in accidents at work, the largest decrease was recorded in Greece (−81%). The increase in the number of people injured in accidents at work was recorded in three countries: France (increase in 2017 +18%), Lithuania (+20%), and Hungary (+5%) [5]. The production sector, also in most countries, recorded a reduction in the number of people injured in accidents at work, the leader in this area was also Greece (−89%); moreover, a decrease in the number of injured by at least 50% was recorded in the Czech Republic, Denmark, Spain, Italy, Malta, The Netherlands, and Finland. The increase in the number of people injured in accidents at work in the manufacturing sector was recorded in Latvia (+38%), Lithuania (+28%), Great Britain (+20%), Ireland (+13%), and Estonia (+2%). The number of fatalities in accidents at work in the country (Table 3) also decreased in most countries, the most in Poland (−59%), The Netherlands (−58%), Lithuania (−52%), and Slovakia (−50%). Unfortunately, this number increased in four countries, in France by over 100%, and in Luxembourg (+60%), Great Britain (+59%), and Croatia (+26% as compared to 2010). The number of fatalities in the production sector has also decreased in most countries, especially, as shown in percentages, in Ireland and Sweden (at least −70%) and in absolute terms in Italy (86 fewer deaths). In three countries (Denmark, France, and Great Britain), there was an increase in the number of fatalities in accidents at work, the most in France—an increase by 70% (32 victims). 

The RR for each country, as determined in each year, made it possible to study the trend of RR changes in 2008–2018. Table 2 presents the statistically significant (*p* < α) results of the Spearman’s rank correlation coefficient significance test (r Spearman). Countries for which a monotonic increase (positive correlation) or decrease (negative correlation) of RR were observed were indicated.

The above data indicate, in most cases, the effectiveness of the pro-safety policy in the EU, the visual effects of which are OSHA campaigns. In recent years, OSHA campaigns have focused on: hazardous substances (2018–2019), health and safe working conditions from the beginning to the end of an employee’s life (2016–2017), or stress at work (2014–2015) [16]. The exceptions are two countries, Great Britain and especially France, where, in most cases, an increase in accident rates was recorded.

### 3.1. The Risk of an Accident at Work and the GDP of EU Countries

Table 1 presents the relative risk of an accident at work in all EU countries. The obtained results indicate a group of countries with a definitely lower risk of an accident than the rest of the EU, these are Romania and Bulgaria. The RR of accident at work in these countries in relation to the entire EU is in the range of 0.03–0.05. Such a low level potentially indicates that workers that are employed in other EU countries suffer from accidents at work several dozen times more often than in Romania and Bulgaria. These two countries are the poorest countries in the European Union (GDP/person in 2018 in Bulgaria is 9,314 and in Romania is USD 12,270) [16]. Almost 70% of GDP is produced in the private sector. In Romania, unemployment calculated for the entire country does not exceed 10%, although, in some regions, it even reaches 60% [5]. The most important industries in Romania are mining, metallurgy and the machine industry. However, in Bulgaria, there are copper deposits and small iron ore. The production of steel, copper, zinc, and lead is dynamic, as well as machinery and equipment, chemicals, and food industry products, which provide most of the income from exports. Tourism is growing in both Bulgaria and Romania. The above does not indicate the development of sectors that do not generate many accidents at work (e.g., banking [5]), which implies questioning the reliability of the accident data reported to Eurostat. Therefore, these countries were left out of the discussion of the results. The country in which RR of an accident at work is the highest in relation to the EU’s average is Luxembourg (RR [95% Cl] = 1.99 [1.88–2.11]), whose GDP is over $115 thousand USD per person [2]. Table 3 presents the relationship between the RR of the analysed countries and the GPD.

The above list shows the existence of a tendency of an increase in the accident risk along with an increase in the GDP of a given country. Initially, it could be stated that countries with higher GDP have a greater risk of injury, but other evidence [19] suggests that countries with a higher GDP tend to have a more highly evolved safety culture. This culture is shaped by large corporations, which, while increasing the level of safety, also implement programmes recording a larger number of accident incidents, which, in countries with lower GDP, are ignored in statistics. However, this hypothesis requires further research. Sweden and the United Kingdom are derogations from the above-mentioned thesis, with relatively high GDP and low RR. Assuming that these countries register a greater number of incidents than countries with lower GDP, it should be stated that the level of work safety in Sweden and Great Britain is higher than in other countries. The situation is different in the case of Spain and Portugal, where the RR of an accident at work is high and GDP relatively low. This may indicate a relatively lower level of safety in Spain and Portugal.

### 3.2. The Risk of an Accident at Work in the EU Countries in the Manufacturing Sector in 2008–2018

**Figure 4:**
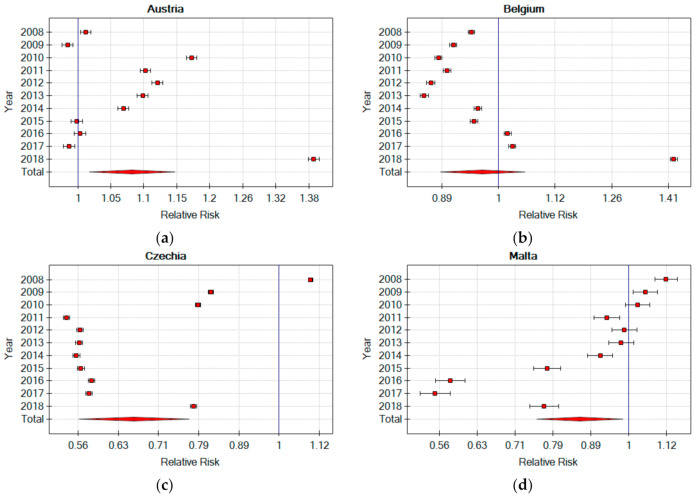
Relative risk of an accident at work in selected EU countries in relation to the risk in the EU in 2008–2018 (own elaboration) (**a**) Austria Relative Risk (**b**) Belgium Relative Risk (**c**) Czechia Relative Risk (**d**) Malta Relative Risk.

Sustainable development is one of the foundations of the EU functioning [20,21]. The development of techniques and technologies, as well as work organisation and the implementation of the Industry 4.0 concept [22,23], during the analysed 10-year period, were confronted with the global economic crisis, and, above all, with the economic crisis experienced by Greece [24]. All of this could disrupt the sustainable development of EU member countries, also in the area of work safety. When analysing the change in RR for the occurrence of an accident at work for individual EU countries in 2008 and 2018, no significant differences were found in the order of individual countries, assuming an accident at work RR as the criterion. However, it is worth noting that the RR of two countries, Malta and Czech Republic, has fallen below the EU average in 10 years (RR < 1), while, in the case of two countries (Austria and Belgium), the RR has increased above 1 (Figure 4). In order to confirm the trend, it is necessary to observe the indicators for these countries in the following years. Significant reduction in RR has also been observed in Greece, but it may not be caused by the improvement in safety in plants, but rather by the increase in unemployment resulting from the crisis [24] and the liquidation of numerous social programmes that discouraged workers from reporting minor injuries at work.

In 2018, the European Union brought together 28 Member States, covering an area of over four million km^2^. The development of the economies of individual Member States, although regulated by treaties and implementing acts, is also conditioned by their geographic location, access to raw materials and the policy of each country. Because of the above, it is very diverse. This differentiation, and hence the unsustainable development of individual countries, is determined by the differentiation of the RR index for registered accidents at work in the production sector of individual countries compared to the RR of the entire EU in 2008–2018 (Figure 1). This index ranges from RR [95% Cl] = 0.195 [0.151–0.252] for Latvia to RR [95% Cl] = 3.288 [2.927–3.649] for Luxembourg. However, it should be remembered that Latvia is inhabited by less than 2 million people and Luxembourg is inhabited by slightly more than 0.6 million, which is only a fraction of the total population of the EU (approx. 450 million [4]). Taking into account all of the results obtained, as presented in Figure 1, we can distinguish three groups of countries with RR in the industrial sector:

- lower than the EU’s RR (Ireland, Greece, Croatia, Cyprus, Latvia, Lithuania, Hungary, Poland, Slovakia, Sweden, and United Kingdom),
- similar to RR across the EU (Belgium, Czech Republic, Estonia, and Italy), and
- higher than the EU’s RR (Denmark, Germany, Spain, France, Luxembourg, Malta, The Netherlands, Austria, Portugal, Slovenia, and Finland).

**Figure 5:**
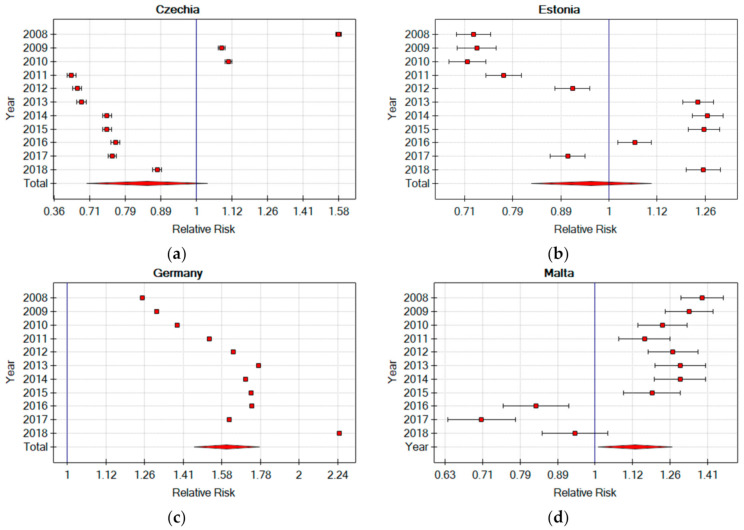
Relative risk of an accident at work in the production sector in selected EU countries in relation to the risk in the EU industrial sector in 2008–2018 (own elaboration). (**a**) Czechia Relative Risks (**b**) Estonia Relative Risk (**c**) Germany Relative Risk (**d**) Malta Relative Risk.

When analysing the obtained results, it was found that, in the group of countries with a lower RR in the production sector than the EU’s average in this sector, only four countries (Ireland, Greece, Sweden, and GB) joined the EU before 2000, while Croatia, Hungary, and Poland constitute three of the four member countries whose currency is not the euro. In the group of countries with a higher RR in relation to the EU’s average in the industrial sector, only two countries (Malta and Slovenia) joined the EU after 2000, and the euro is the currency of all countries in this group. It was found that, between 2008–2018, in two countries (Czech Republic and Malta) the RR level in relation to the EU decreased from more than 1 to less than 1, and in one case (Estonia) the RR level in relation to the EU increased from less than 1 to more than 1. Of the three mentioned above countries, the largest difference in the RR value was recorded in the Czech Republic (−0.71). However, among all of the EU countries, the largest difference in the RR value in the production sector was recorded in Germany, an increase of 1.00 (Figure 5). All of the above results may seemingly indicate that, in the so-called “old” EU, the level of worker safety in the production sector is much lower than in this sector in the so-called “new” EU. However, in fact, these results may indicate differences in the approach to safety and accident recording in individual countries due to differences in national and organizational culture [25,26]. 

The concept of Industry 4.0 [23], promoted and implemented in recent years, announced by the German Chancellor, based on, inter alia, the issue of BigData, assumes that the user/decision maker has virtually unlimited access to all information resources, including those that are related to all accidents at work [27]. Such operation of IT systems results in greater data reliability. Countries that were incorporated into the EU in the 21st century, despite meeting a number of economic conditions, are still going through a period of transformation, especially in the industrial sector, which, in most of these countries, is striving for the assumptions of Industry 3.0, based on automation, which leaves data reliability dependent on the human factor [28]. Among the countries mentioned, Sweden deserves attention in the context of striving for sustainable development of the EU, as it constitutes the 6th EU economy in terms of GDP. Sweden’s low RR in the industrial sector indicates a high safety culture in the sector, which should set standards for other countries [29,30,31].

The size and development of the manufacturing sector depend on many geopolitical factors. However, assuming that this sector, due to the production processes implemented, is, by definition, an accident-prone sector, it should be expected that the RR level of an accident at work compared to the average risk in the country will always be higher. The results of the analysis in this regard are presented in Figure 2. Overall, for the entire EU, the RR in the manufacturing sector in 2008–2018 is RR [95% Cl] = 1.397 [1.331–1.466], while, in 2014–2017, this indicator did not exceed 1.3. The countries in which the RR value of an accident at work in the industrial sector did not differ from the national RR are France and Ireland. Countries in which the RR of an accident in the industrial sector is at least double when compared to others are: the Czech Republic, Estonia, Greece, Cyprus, Latvia, Lithuania, Luxembourg, Hungary, Poland, Romania, and Slovakia. It should be noted that eight of the above countries joined the EU after 2000. The higher RR for these countries confirms the previously presented hypothesis regarding the need for intensive development of domestic industry among the so-called “new” EU towards Industry 4.0. The highest RR in this respect was recorded for Greece RR [95% Cl] = 3.007 [2.755–3.281], which could have resulted from the deep economic crisis that this country experienced in the analysed period [24]. Taking into account the size of the economies of individual member countries, it was stated that the three largest economies in the EU: German, French, and Italian, generating about 50% of the EU GDP, employing over 15 million people [5], are the most sustainable economies in the EU from the point of view of work safety, because the RR of accident in the industrial sector in these countries, in relation to the national data, is one of the lowest in the entire EU, not exceeding 1.3.

### 3.3. The Risk of Fatal Accidents in the Industrial Sector of EU Countries

**Figure 6:**
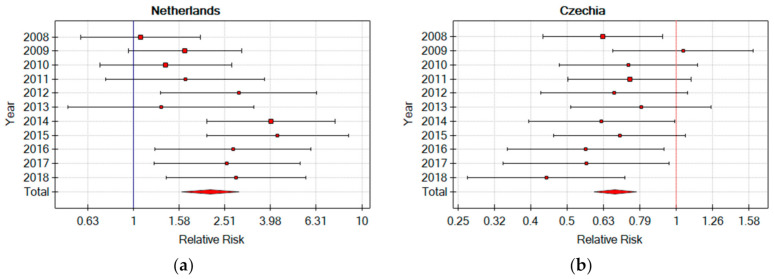
Relative risk of a fatal accident at work in the production sector in The Netherlands and Czechia in relation to the risk of a fatal accident at work in 2008–2018 (own elaboration) (**a**) Netherlands Relative Risks (**b**) Czechia Relative Risk.

The number of fatal accidents at work constitutes the most tragic indicator of safety in a sector or country. Figure 3 presents the results of the RR analysis of the fatal accident in the production sector in relation to the RR of the fatal accident in each of the EU countries in 2008–2018. The results are characterised by a large Clopper–Pearson confidence interval due to the relatively low number of fatal accidents at work [32]. The obtained results indicate that in 12 EU countries, the RR of a fatal accident in the production sector is not statistically significantly different from the RR of such an accident in the whole country. The lowest RR of fatal accidents in the production sector occurs in the Czech Republic and Hungary, not exceeding 0.7, while the highest in Greece and The Netherlands (RR over 2.1). This is particularly important in The Netherlands, which constitutes the 5th economy of the EU, where 9–10% of citizens are employed in the production sector, and about 11–31% of all fatal accidents occur in the following years. The Netherlands is a leading natural gas producing country, so the energy sector should be based on sustainable development, also in health and safety [33]. In the case of The Netherlands, an increase was observed with RR [95% Cl] = 1.077 [0.590–1.963] (*p* = 0.810) in 2008 to RR [95% Cl] = 2.821 [1.397–5.696] (*p* = 0.004) in 2018 (Figure 6). A particularly noticeable increase in the percentage of fatalities of accidents at work in The Netherlands has been recorded since 2014, where it accounts for at least 20% of the total number of victims in the country [5]. For comparison, in the largest economy of the EU, the German, in the industrial sector, with 19–21% employment, the share of all fatal accidents in the analysed years was 14–17%.

### 3.4. Limitations and Directions for Future Research

The results that are presented in the paper are based on Eurostat data [5]. The quality of data depends on the reliability of reporting all accidents at work in each country. For some countries, the data provided are questionable, as shown in the text. In Greece and Romania, only 5% of all EU workers are employed, which does not significantly affect the results of the presented analysis. The data from these countries had to be included in the analysis in order to show the entirety of changes in the EU. Further research should focus on identifying the causes of poor-quality data reported in some countries and developing a program for their improvement, for example, by defining the same reporting standards in all countries. For example, the definition of an accident at work varies from country to country.

The results that were presented in the article made it possible to identify countries in which there were positive and negative changes in accidents at work in the manufacturing sector. Further research should be aimed at identifying good practices in accident prevention in countries with positive changes. This practice can be an example for countries with poorer RR. Further research should also focus on identifying the causes of incidental changes in RR in particular years, e.g., in Austria and Belgium in 2018 (Figure 4). This will allow the EU to more sustainably develop in the area of occupational safety.

## 4. Conclusions

The conducted analysis allowed the formulation of the following conclusions:

- in most EU countries, in the years 2008–2018 there was a decrease in the number of people injured in accidents at work and in fatal accidents,
- Sweden’s low RR in the industrial sector indicates a high safety culture in the sector, which should set standards for other countries,
- the value of the RR indicator increases with the increase in the country’s GDP, which can be explained by the increased reliability of the related data to the improvement of organisational culture and investments that are related to the implementation of Industry 4.0, and
- the RR in the manufacturing sector of the so-called “old” EU is higher than in the so-called “new” EU, which may result from the implementation of Industry 4.0 assumptions in the “old” EU countries, which, based on Big Data, collect all data on accidents at work. In countries where the industry is based on the principles of Industry 3.0, the collected accident data depend on the human factor.

The quality of the presented studies depends on the quality of the available data. The obtained results show that not all countries attach equal importance to the reliability of data that are transmitted to Eurostat. The presented results and conclusions may be useful in shaping the EU’s policy in the field of sustainable development of the production sectors of individual member countries, because the literature on the subject lacks comprehensive research in this field, concerning all EU countries.

## Figures and Tables

**Table 1:** Relative risk of an accident at work in the EU countries in relation to the risk in the EU in 2008–2018 (own elaboration).

Country	Relative Risk (RR)	SE (ln)	−95%CI	+95%CI	*p*-Value
Austria	1.079	0.030	1.017	1.145	0.012
Belgium	0.969	0.043	0.890	1.055	0.465
Bulgaria	0.052	0.030	0.049	0.055	<0.001
Croatia	0.572	0.044	0.525	0.623	<0.001
Cyprus	0.338	0.046	0.308	0.370	<0.001
Czechia	0.660	0.080	0.564	0.773	<0.001
Denmark	1.381	0.029	1.306	1.460	<0.001
Estonia	0.661	0.044	0.606	0.720	<0.001
Finland	1.257	0.026	1.193	1.323	<0.001
France	1.711	0.071	1.490	1.965	<0.001
Germany	1.670	0.042	1.538	1.814	<0.001
Greece	0.135	0.153	0.100	0.182	<0.001
Hungary	0.344	0.050	0.312	0.379	<0.001
Ireland	0.551	0.060	0.490	0.620	<0.001
Italy	1.054	0.045	0.966	1.150	0.240
Latvia	0.121	0.090	0.101	0.144	<0.001
Lithuania	0.154	0.084	0.130	0.181	<0.001
Luxembourg	1.992	0.030	1.878	2.112	<0.001
Malta	0.862	0.066	0.757	0.982	0.025
The Netherlands	0.991	0.091	0.829	1.186	0.922
Poland	0.333	0.030	0.314	0.353	<0.001
Portugal	1.862	0.028	1.764	1.967	<0.001
Romania	0.031	0.064	0.027	0.035	<0.001
Slovakia	0.259	0.039	0.240	0.279	<0.001
Slovenia	0.922	0.092	0.770	1.103	0.374
Spain	1.737	0.048	1.582	1.907	<0.001
Sweden	0.329	0.147	0.247	0.438	<0.001
United Kingdom	0.495	0.025	0.471	0.521	<0.001

Cl—Clopper-Pearson confidence interval.

**Table 2:** Monotonicity of changes in relative risk of an accident at work in the EU countries in 2008–2018 (own elaboration).

	Positive Correlation	Negative Correlation
	Country (r Spearman)	Country (r Spearman)
RR of an accident at work in the EU countries in relation to the risk in the EU in 2008–2018	Belgium (0.682); Croatia (0.886); Estonia (0.791); France (0.976); Germany (0.764); Hungary (0.755); Latvia (0.945); Lithuania (0.964); Portugal (0.903); Romania (0.764); Slovakia (0.627); Sweden (0.700)	Greece (−0.809); Italy (−0.664); Malta (−0.945); The Netherlands (−0.645)
RR of an accident at work in the production sector in EU countries in relation to the risk in the EU industrial sector in 2008–2018	Bulgaria (0.655); Croatia (0.986); Estonia (0.709); France (0.806); Germany (0.809); Hungary (0.836); Ireland (0.727); Latvia (0.964); Lithuania (0.973); Portugal (0.755); Slovakia (0.673); Sweden (0.973); UK (0.845)	Greece (−0.845); Italy (−0.718); Malta (−0.773)
RR of an accident at work in the production sector of the EU countries in relation to the risk of an accident at work in the country in the years 2008–2018	Ireland (0.627); Latvia (0.882); Lithuania (0.673); Luxembourg (0.691); The Netherlands (0.664); UK (0.800)	Belgium (−0.736); Czechia (−0.891); Cyprus (−0.618); Denmark (−0.764); Finland (−0.945); France (−0.976); Germany (−0.627); Greece (−0.982); Spain (−0.845); Poland (−0.918); Portugal (−0.879); Romania (−0.827); Slovakia (−0.773); Slovenia (−0.709); Sweden (−0.636)
RR of a fatal accident at work in the production sector in the EU countries in relation to the risk of a fatal accident at work in the country in 2008–2018	The Netherlands (0.618)	Czechia (−0.691)

**Table 3:** The risk of an accident at work in EU countries (according to GDP).

RR	Country	GPD/Person, Thousand USD [2]
0.03–0.05	Bulgaria *, Romania *	9.3–12.3
0.12–0.15	Greece, Latvia *, Lithuania *	15–37 (without Sweden)
0.26–0.34	Cyprus *, Hungary *, Poland *, Slovakia *, Sweden	
0.50–0.66	Croatia *, Czechia *, Estonia *, Great Britain, Ireland, Malta * (0.86)	20–40 (without Great Britain)
about 1	Austria, Belgium, Italy, The Netherlands, Slovenia *	27–50
1.26–1.38	Denmark, Finland, France, Germany, Portugal, Spain	31–56
1.99	Luxembourg	115

* the so-called countries of the “new” EU.

## Data Availability

Not applicable.
